# Incobotulinumtoxin A for Sialorrhea in Neurological Disorders: A Real-Life Experience

**DOI:** 10.3390/toxins10060217

**Published:** 2018-05-28

**Authors:** Javier Martínez-Poles, Velina Nedkova-Hristova, José Bernardo Escribano-Paredes, Sebastián García-Madrona, Elena Natera-Villalba, Carlos Estévez-Fraga, José Luis López-Sendón Moreno, Icíar Avilés-Olmos, Gema Sánchez Díaz, Juan Carlos Martínez Castrillo, Araceli Alonso-Canovas

**Affiliations:** 1 Department of Neurology, Hospital Universitario Ramón y Cajal, 28034 Madrid, Spain; velinanh@hotmail.com (V.N.-H.); bernardoescribano87@gmail.com (J.B.E.-P.); sebas.gmadrona@gmail.com (S.G.-M.); elena.natera.v@gmail.com (E.N.-V.); cefraga@alumni.unav.es (C.E.-F.); joselopezsendon@hotmail.com (J.L.L.-S.M.); iavilesolmos@gmail.com (I.A.O.); gsanchez@salud.madrid.org (G.S.D.); jmcastrillo@salud.madrid.org (J.C.M.C.); aracelialcan@yahoo.es (A.A.C.); 2 Department of Neurodegenerative Diseases, UCL Institute of Neurology, London WC1N 3RX, UK

**Keywords:** sialorrhea, neurological disorders, Parkinson’s disease, botulinum toxin type A, incobotulinumtoxin A

## Abstract

Botulinum toxin type A is one of the most useful treatments of sialorrhea in neurological disorders. Evidence for the use of incobotulinumtoxin A (inco-A) in the treatment of sialorrhea is limited. Thirty-six patients with sialorrhea were treated with infiltrations of inco-A into both parotid glands. The severity of sialorrhea was evaluated by the Drooling Severity Scale (DSS), and the Drooling Frequency Scale (DFS). Patients’ perceptions of clinical benefit were recorded via the Patient Global Impression of Improvement (PGI-I) scale. Following treatment, there was a significant difference in both the DFS and the DSS (*p* < 0.001). Clinical benefits on the basis of the PGI-I were present in up to 90% of patients.

## 1. Introduction

Sialorrhea is a frequent and disabling symptom of neurological disorders that is present in about 10% of patients with chronic neurological disorders, and in nearly 80% of Parkinson’s disease (PD) patients [1,2,3,4]. The etiology of sialorrhea in patients with neurodegenerative disorders is probably multifactorial. Many studies showed that PD patients produced less saliva when compared with normal controls [5]. Open-mouth posture, discoordination between oral and pharyngeal stages of swallowing, reduction of spontaneous swallowing, flexed posture, or limited tongue movements can all contribute to disturbances in intraoral saliva management, and sialorrhea [6,7,8]. In PD, there is evidence that the severity of sialorrhea is correlated with dysphagia [7]. Sialorrhea is an important non-motor symptom because it may cause silent aspiration with secondary respiratory infections [9,10], and has a major impact on quality of life [11].

Botulinum toxin (BoNT) is an established and effective treatment for sialorrhea, blocking the release of acetylcholine at the parasympathetic terminals of the salivary glands [12]. There is currently evidence for the efficacy of onabotulinumtoxin A (ona-A) [13,14], abobotulinumtoxin A [15,16], and botulinum toxin type B [17,18,19] as treatments for sialorrhea, with no differences between them, but there are no studies to support the efficacy of incobotulinumtoxin A in treating sialorrhea in neurological diseases [20,21].

Incobotulinumtoxin A (inco-A, Xeomin^®^, Merz Pharmaceuticals, Frankfurt, Germany) is a purified BoNT type A (BoNT-A) that contains botulinum neurotoxin without complexing proteins; thus, the formation of neutralizing antibodies is lower than with other BoNT [22]. It can also be stored at room temperatures <25 °C [23]. Routine use of inco-A showed efficacy and safety in a variety of disorders [22,24,25,26]. The use of inco-A for the treatment of sialorrhea in neurological diseases is controversial, with some studies showing efficacy [27], and others showing no significant differences with a placebo [28].

## 2. Results

Thirty-six patients with disabling sialorrhea were included in the study. The mean age of the sample was 71.1 ± 17.9 years old. 63.9% of patients were male. Movement disorders (PD, atypical parkinsonism, and Huntington’s disease) were the most frequent diagnosis in the sample (77.8%). Other diagnoses included encephalopathy, Alzheimer’s disease, and cerebrovascular disease (Table 1). Dilution was made with 1 mL of saline solution for 100 international units (IU).

During the follow-up, patients received an average of 2.3 ± 1.1 infiltrations of inco-A. The duration of the effect (considered as the time of clinical benefit with the optimal dose of inco-A) was 4.4 ± 2.0 months. An average of 43.2 ± 7.6 IU of inco-A (43.2 ± 7.6 mouse LD_50_) was injected into each parotid gland. It was necessary to increase the initial dose of inco-A in eight patients (22.2%) due to partial efficacy.

Twenty-three patients (63.9%) were infiltrated in two community-based outpatient Movement Disorders Clinics, while the remaining 13 (36.1%) were injected in our tertiary hospital outpatient Movement Disorders Clinic. The peculiarity of the community-based clinics was the absence of refrigerating conditions for drug storage, making inco-A the only toxin available for injections in this setting. Patients that followed up in this community-based clinic tended to have more mobility problems and more advanced diseases, and benefited from the accessibility and proximity of these clinics as opposed to the farther tertiary center.

Five patients (13.9%) had previously received other BoNTs (three of them with BoNT-B and two of them ona-A), and a change to inco-A was decided due to their lack of efficacy.

The Drooling Severity Scale (DSS), the Drooling Frequency Scale (DFS), and the Patient Global Impression of Improvement (PGI-I) scale were registered in 32 of 36 patients, while the remaining four patients did not attend the follow-up visits.

**Figure 1:**
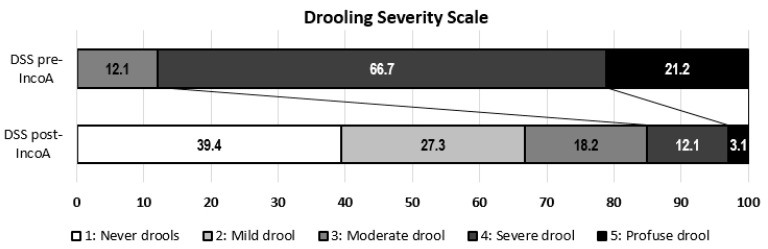
Drooling Severity Scale score before and after the administration of incobotulinumtoxin A (inco-A).

**Figure 2:**
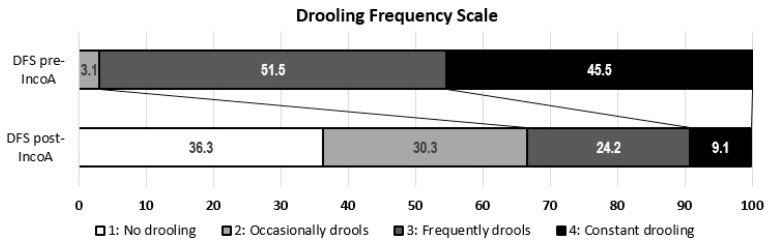
Drooling Frequency Scale score before and after the administration of inco-A.

After the infiltration of inco-A, there were significant improvements in the DSS (2.1 ± 1.2 vs. 4.1 ± 0.6, *p* < 0.001) and the DFS (2.1 ± 1.0 vs. 3.4 ± 0.6, *p* < 0.001) (Figure 1 and Figure 2).

**Figure 3:**
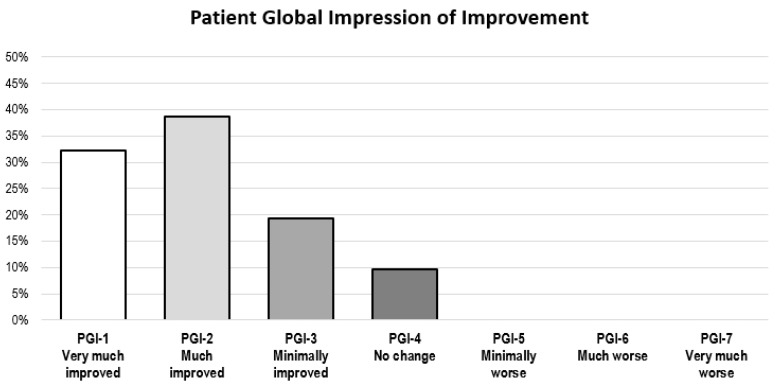
Patient Global Impression of Improvement Scale after the administration of inco-A.

As for the PGI-I scale, 29 of 32 patients (90.6%) reported improvement after inco-A administration (PGI-I < 4), with 23 of them (71.9%) reporting much or very much improvement (PGI-I ≤ 2) (Figure 3). There was no significant difference in responses between PD patients and non-PD patients (*p* = 0.751).

The 29 patients who perceived clinical benefits (responders) needed an average of 43.8 ± 7.7 IU of inco-A into each parotid gland, while the three patients without clinical benefits needed an average of 38.3 ± 2.9 IU, although this difference was not statistically significant (*p* = 0.209).

**Figure 4:**
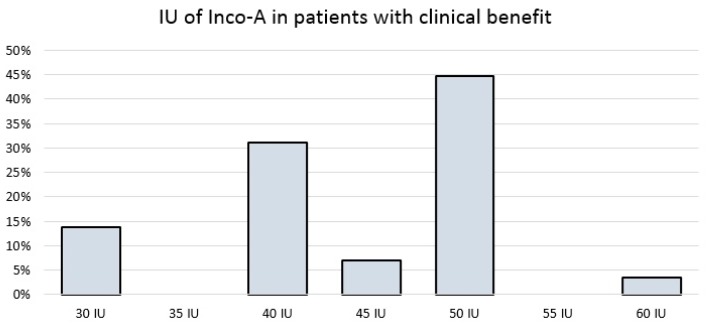
International Units of inco-A in patients who perceived clinical benefits.

Among the 29 responders, most patients (24, 82.8%) required between 40 IU and 50 IU, while four patients (14.3%) required 30 IU or less, and one patient required the maximum dose of 60 IU because of the incomplete effect of inco-A at lower doses (Figure 4).

Four of the five patients (80%) who had received previous treatment of sialorrhea with other BoNTs improved with inco-A, although, in one of them, improvement was perceived as mild. There was no difference between de novo patients and those who had previously received other BoNTs (*p* = 0.394).

As for adverse effects, only one patient (2.8%) reported mild and transitory worsening of previous dysphagia, two months after infiltration with inco-A. No other adverse effects were reported.

## 3. Discussion

Our findings supported the efficacy and safety of inco-A in treating patients with sialorrhea in neurological diseases, mainly PD, with similar results to those found with other types of BoNTs [1]. Our data were consistent with the comparable efficacy of inco-A to other subtypes of BoNTs in various pathologies, such as spasticity, dystonia, blepharospasm, or hemifacial spasm [23,29,30,31]. To be noted, the most frequently used amount of inco-A in our study (50 IU into each parotid gland) was the same as the amount used with other BoNTs such as ona-A in other studies, with similar results (88% of patients with clinical benefits with ona-A in a previous study versus 90.6% in our study with inco-A) [13]. A recent report showed benefits in patients with neurological disorders receiving BoNT-A (ona-A and inco-A), with better responses in those injected in 4 glands (two parotid glands and two submandibular glands) [27]. Only one study unsuccessfully attempted to demonstrate the efficacy of inco-A in the treatment of sialorrhea [28], possibly due to a different pattern of injection, where only 20 IU of inco-A was infiltrated into each parotid gland, and 30 IU into each submandibular gland.

In our study, we obtained clinical benefits when only the parotid glands were addressed. This may be because most patients in our series were patients with PD. Patients with PD do not have an increase in the basal production of saliva, the secretion of which the submandibular glands contribute to the most. However, they have poor management of secretions, and hypersalivation due to stimuli, the secretion of which the parotid glands contribute to the most [32]. Nonetheless, as shown in a recent study [27], patients may benefit from additional injections into the submandibular glands in cases of a lack of response.

Finally, a significant proportion of patients (64%) enjoyed an additional benefit of inco-A treatment for sialorrhea. Due to a lack of the need of refrigeration for drug storage, treatment with inco-A was feasible in our community-based outpatient Movement Disorders Clinics, which are generally aimed at patients with advanced diseases and more severe problems of mobility, restricting their periodic follow-ups in the less accessible and farther tertiary center.

Our study had some limitations. It was a retrospective and uncontrolled study, based on clinical history data, so it may have had the biases derived from this type of study. In addition, the variables used to measure the effectiveness of the study were all subjective, although they were widely used in studies on sialorrhea, and were validated [33,34]. The sample size of our study was also limited, although it was similar to previous studies in this area [18].

## 4. Conclusions

In our experience, inco-A is an effective and safe treatment for sialorrhea, and could be considered as an alternative to other BoNTs.

## 5. Materials and Methods

In this retrospective study, we included 36 patients with neurological disorders and disabling sialorrhea, treated with inco-A in our tertiary hospital Movement Disorders (MD) Unit, and its two community-based outpatient Movement Disorders Clinics between 1 January 2017 and 31 October 2017. Infiltrations with inco-A into both parotid glands were performed in all patients with guidance from anatomic landmarks. Our MD Unit’s protocol for sialorrhea treatment was followed. Only parotid glands were considered for injections for safety reasons (higher likelihood of dysphagia in submandibular glands), and due to an overall good response to parotid-only injections in our experience. Starting doses were calculated ranging from 30 IU to 50 IU, taking into account body mass index and severity of sialorrhea. Whenever response was satisfactory, the same schedule was maintained in further injections. In the cases of suboptimal or no response to the first set of injections, a higher dose was injected. The increase in dosage was left to the clinician’s judgment, and generally reached 50 UI per parotid gland.

Efficacy was assessed by changes in score in the Drooling Severity Scale (DSS) and the Drooling Frequency Scale (DFS). DSS classifies severity of drooling using a five-level domain, ranging from 1 (dry) to 5 (profuse drooling). DFS classifies frequency of sialorrhea using a four-level domain, ranging from 1 (no drooling) to 4 (constant drooling). (Table 2). The best responses to inco-A dosage were registered.

The clinical benefits perceived by the patients were assessed using the Patient Global Impression of Improvement (PGI-I) scale (Table 3).

## Figures and Tables

**Table 1:** Demographic characteristics and clinical diagnosis.

**Demographic characteristics**	**Mean ± SD/N (%)**
Age	71.1 ± 17.9 years
Sex, male	23 (63.9%)
Previous treatment with other BoNT	5 (13.9%)
Botulinum toxin B	3 (8.3%)
Onabotulinumtoxin A	2 (5.6%)
**Clinical diagnosis**	**N (%)**
Parkinson’s disease	21 (58.3%)
Atypical Parkinsonism	6 (16.7%)
Encephalopathy	5 (13.9%)
Post-traumatic encephalopathy	2 (5.6%)
Connatal encephalopathy	2 (5.6%)
Epileptic encephalopathy	1 (2.8%)
Alzheimer’s disease	2 (5.6%)
Cerebrovascular disease	1 (2.8%)
Huntington’s disease	1 (2.8%)

**Table 2:** Drooling Severity and Frequency Scales.

**Severity**	
1	Dry (never drools)
2	Mild drooling, only lips wet
3	Moderate drooling, drool reaches the lips and the chin
4	Severe drooling; drool drips off chin and onto clothing
5	Profuse drooling, drooling off the body and onto objects
**Frequency**	
1	No drooling
2	Occasionally drools
3	Frequently drools
4	Constant drooling

**Table 3:** Patient Global Impression of Improvement scale.

1	Very much improved
2	Much improved
3	Minimally improved
4	No change
5	Minimally worse
6	Much worse
7	Very much worse

## References

[1] Porta M., Gamba M., Bertacchi G., Vaj P. (2001). Treatment of sialorrhoea with ultrasound guided botulinum toxin type A injection in patients with neurological disorders. J. Neurol. Neurosurg. Psychiatry.

[2] Kalf J.G., De Swart B.J.M., Borm G.F., Bloem B.R., Munneke M. (2009). Prevalence and definition of drooling in Parkinson’s disease: A systematic review. J. Neurol..

[3] Politis M., Wu K., Molloy S., Bain P.G., Chaudhuri K.R., Piccini P. (2010). Parkinson’s disease symptoms: The patient’s perspective. Mov. Disord..

[4] Lakraj A.A., Moghimi N., Jabbari B. (2013). Sialorrhea: Anatomy, pathophysiology and treatment with emphasis on the role of botulinum toxins. Toxins.

[5] Srivanitchapoom P., Pandey S., Hallett M. (2014). Drooling in Parkinson’s disease: A review. Parkinsonism Relat. Disord..

[6] Proulx M., de Courval F.P., Wiseman M.A., Panisset M. (2005). Salivary production in Parkinson’s disease. Mov. Disord..

[7] Nóbrega A.C., Rodrigues B., Torres A.C., Scarpel R.D., Neves C.A., Melo A. (2008). Is drooling secondary to a swallowing disorder in patients with Parkinson’s disease?. Parkinsonism Relat. Disord..

[8] Merelh M. (2008). Sialorrhoea and Drooling in Patients with Parkinson. Drugs Ageing.

[9] Rodrigues B., Nóbrega A.C., Sampaio M., Argolo N., Melo A. (2011). Silent saliva aspiration in Parkinson’s disease. Mov. Disord..

[10] Nóbrega A.C., Rodrigues B., Melo A. (2008). Is silent aspiration a risk factor for respiratory infection in Parkinson’s disease patients?. Parkinsonism Relat. Disord..

[11] Kalf J.G., Smit A.M., Bloem B.R., Zwarts M.J., Munneke M. (2007). Impact of drooling in Parkinson’s disease. J. Neurol..

[12] Tan E.K. (2006). Botulinum Toxin Treatment of Sialorrhea: Comparing Different Therapeutic Preparations. Eur. J. Neurol..

[13] Lagalla G., Millevolte M., Capecci M., Provinciali L., Ceravolo M.G. (2006). Botulinum toxin type A for drooling in Parkinson’s disease: A double-blind, randomized, placebo-controlled study. Mov. Disord..

[14] Sillanpaä S., Sipilä M., Numminen J., Rautiainen M. (2015). The Experience of Treating Drooling with Repeated Botulinum Toxin Injections. ORL J. Otorhinolaryngol. Relat. Spec..

[15] Mazlan M., Rajasegaran S., Engkasan J.P., Nawawi O., Goh K.J., Freddy S.J. (2015). A double-blind randomized controlled trial investigating the most efficacious dose of botulinum toxin-A for sialorrhea treatment in asian adults with neurological diseases. Toxins.

[16] Weikamp J.G., Schinagl D.A.X., Verstappen C.C.P., Schelhaas H.J., de Swart B.J.M., Kalf J.G. (2016). Botulinum toxin-A injections vs. radiotherapy for drooling in ALS. Acta Neurol. Scand..

[17] Chinnapongse R., Gullo K., Nemeth P., Zhang Y., Griggs L. (2012). Safety and efficacy of botulinum toxin type B for treatment of sialorrhea in Parkinson’s disease: A prospective double-blind trial. Mov. Disord..

[18] Ondo W.G., Hunter C., Moore W. (2004). A double-blind placebo-controlled trial of botulinum toxin B for sialorrhea in Parkinson’s disease. Neurology.

[19] Lagalla G., Millevolte M., Capecci M., Provinciali L., Ceravolo M.G. (2009). Long-lasting benefits of botulinum toxin type B in Parkinson’s disease-related drooling. J. Neurol..

[20] Guidubaldi A., Fasano A., Ialongo T., Piano C., Pompili M., Mascianà R., Siciliani L., Sabatelli M., Bentivoglio A.R. (2011). Botulinum toxin A versus B in sialorrhea: A prospective, randomized, double-blind, crossover pilot study in patients with amyotrophic lateral sclerosis or Parkinson’s disease. Mov. Disord..

[21] Sridharan K., Sivaramakrishnan G. (2018). Pharmacological interventions for treating sialorrhea associated with neurological disorders: A mixed treatment network meta-analysis of randomized controlled trials. J. Clin. Neurosci..

[22] Dressler D. (2012). Five-year experience with incobotulinumtoxinA (Xeomin(®)): The first botulinum toxin drug free of complexing proteins. Eur. J. Neurol..

[23] Dressler D., Bigalke H. (2017). Long-term stability of reconstituted incobotulinumtoxinA: How can we reduce costs of botulinum toxin therapy?. J. Neural. Transm..

[24] Dressler D. (2009). Routine use of Xeomin in patients previously treated with Botox: Long term results. Eur. J. Neurol..

[25] Jost W.H., Benecke R., Hauschke D., Jankovic J., Kaňovský P., Roggenkämper P., Simpson D.M., Comella C.L. (2015). Clinical and pharmacological properties of incobotulinumtoxina and its use in neurological disorders. Drug Des. Dev. Ther..

[26] Dressler D., Rychlik R., Kreimendahl F., Schnur N., Lambert-Baumann J. (2015). Long-term efficacy and safety of incobotulinumtoxinA and conventional treatment of poststroke arm spasticity: A prospective, non-interventional, open-label, parallel-group study. BMJ Open.

[27] Restivo D.A., Panebianco M., Casabona A., Lanza S., Marchese-Ragona R., Patti F., Masiero S., Biondi A., Quartarone A. (2018). Botulinum toxin a for sialorrhoea associated with neurological disorders: Evaluation of the relationship between effect of treatment and the number of glands treated. Toxins.

[28] Narayanaswami P., Geisbush T., Tarulli A., Raynor E., Gautam S., Tarsy D., Gronseth G. (2016). Drooling in Parkinson’s disease: A randomized controlled trial of incobotulinum toxin A and meta-analysis of Botulinum toxins. Parkinsonism Relat. Disord..

[29] Benecke R., Jost W.H., Kanovsky P., Ruzicka E., Comes G., Grafe S. (2005). A new botulinum toxin type A free of complexing proteins for treatment of cervical dystonia. Neurology.

[30] Roggenkämper P., Jost W.H., Bihari K., Comes G., Grafe S. (2006). Efficacy and safety of a new Botulinum Toxin Type A free of complexing proteins in the treatment of blepharospasm. J. Neural. Transm..

[31] Akulov M.A., Orlova O.R., Orlova A.S., Usachev D.J., Shimansky V.N., Tanjashin S.V., Khatkova S.E., Yunosha-Shanyavskaya A.V. (2017). IncobotulinumtoxinA treatment of facial nerve palsy after neurosurgery. J. Neurol. Sci..

[32] Proctor G.B. (2016). The physiology of salivary secretion. Periodontology.

[33] Mancini F., Zangaglia R., Cristina S., Sommaruga M.G., Martignoni E., Nappi G., Pacchetti C. (2003). Double-blind, placebo-controlled study to evaluate the efficacy and safety of botulinum toxin type A in the treatment of drooling in parkinsonism. Mov. Disord..

[34] Savarese R., Diamond M., Elovic E., Millis S.R. (2004). Intraparotid Injection of Botulinum Toxin A as a Treatment to Control Sialorrhea in Children with Cerebral Palsy. Am. J. Phys. Med. Rehabil..

